# Novel Motor-Sparing Ultrasound-Guided Neural Injection in Severe Carpal Tunnel Syndrome: A Comparison of Four Injectates

**DOI:** 10.1155/2022/9745322

**Published:** 2022-02-17

**Authors:** Yung-Tsan Wu, King Hei Stanley Lam, Chia-Ying Lai, Si-Ru Chen, Yu-Ping Shen, Yu-Chi Su, Tsung-Ying Li, Chueh-Hung Wu

**Affiliations:** ^1^Department of Physical Medicine and Rehabilitation, Tri-Service General Hospital, School of Medicine, National Defense Medical Center, Taipei, Taiwan; ^2^Integrated Pain Management Center, Tri-Service General Hospital, School of Medicine, National Defense Medical Center, Taipei, Taiwan; ^3^Department of Research and Development, School of Medicine, National Defense Medical Center, Taipei, Taiwan; ^4^The Hong Kong Institute of Musculoskeletal Medicine, Hong Kong; ^5^Department of Family Medicine, The Chinese University of Hong Kong, Hong Kong; ^6^Department of Family Medicine, The University of Hong Kong, Hong Kong; ^7^Center for Regional Anesthesia and Pain Medicine, Wan Fang Hospital, Taipei Medical University, Taipei, Taiwan; ^8^The Taiwan Association of Prolotherapy and Regenerative Medicine, Taichung, Taiwan; ^9^Department of Physical Medicine and Rehabilitation, National Taiwan University Hospital Hsin-Chu Branch, Hsinchu, Taiwan; ^10^Department of Physical Medicine and Rehabilitation, College of Medicine, National Taiwan University, Taipei, Taiwan; ^11^Department of Physical Medicine and Rehabilitation, National Taiwan University Hospital, Taipei, Taiwan

## Abstract

Nerve hydrodissection uses fluid injection under pressure to selectively separate nerves from areas of suspected entrapment; this procedure is increasingly viewed as potentially useful in treating carpal tunnel syndrome (CTS). The usage of normal saline (NS), 5% dextrose water (D5W), platelet-rich plasma (PRP), and hyaluronic acid (HA) as primary injectates for hydrodissection without an anesthetic can limit anesthetic-related toxicity and preserve the motor functions of the median nerve. Here, we describe a novel motor-sparing neural injection and compare the effect of these four injectates for severe CTS. We retrospectively reviewed the outcomes of 61 severe CTS cases after a single neural injection with NS, D5W, PRP, or HA. Outcomes were evaluated on the 1^st^ and 6^th^ months postinjection, including the Boston Carpal Tunnel Questionnaire (BCTQ) scores and the nerve cross-sectional area (CSA). The results revealed that PRP, D5W, and HA were more efficient than NS at all measured time points (*p* < 0.05), except for CSA at the 1^st^ month between the NS and D5W groups. Single-injections of PRP and D5W seemed more effective than that of HA within 6 months postinjection for symptom and functional improvement (6^th^-month BCTQ-symptom, D5W vs. HA, *p* = 0.047; 1^st^-month BCTQ-symptom, PRP vs. HA, *p* = 0.018; 1^st^- and 6^th^-month BCTQ-function, D5W vs. HA, *p* = 0.002 and 0.016, respectively; 1^st^-month BCTQ-function, PRP vs. HA, *p* < 0.001). For reducing CSA, PRP and HA seemed more effective than D5W (HA > PRP > D5W on the 1^st^ month and HA vs. D5W, *p* = 0.001; PRP > HA > D5W on the 6^th^ month and PRP vs. D5W, *p* = 0.012).

## 1. Introduction

Carpal tunnel syndrome (CTS) is the most common entrapment neuropathy [1]. The prevalence of CTS ranged from 1 to 5% with female predominantly [2]. The elderly seem to have a higher prevalence, and studies revealed a bimodal age distribution with the first peak between the ages of 50 and 54 years and a second peak within 75–84 years [3, 4]. The classical symptoms of CTS usually involve in the first three fingers and half of the ring finger with paresthesia or dysesthesia and are often described as a “pin-and-needle” sensation. Nocturnal burning pain is relieved by hand shaking or postural adjustment. In more severe cases, muscle atrophy at the thenar muscles with weakness may present [1].

**Figure 1:**
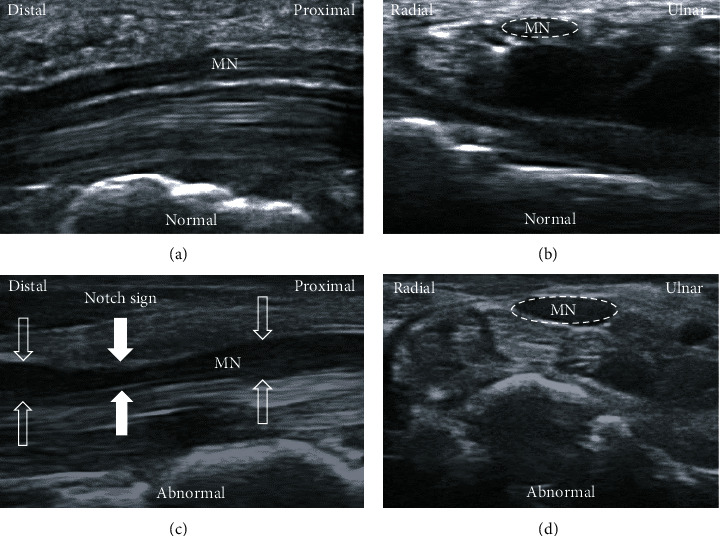
The ultrasonography examination shows normal appearance of median nerve (MN) through carpal tunnel (a) and proximal inlet of carpal tunnel (b). The abnormal findings of MN in carpal tunnel syndrome are shown (c, d). The compression site of median nerve (MN) within carpal tunnel with obvious “notch sign” and swelling of MN proximal and distal to compressive site were observed (c). The segmental increase in the cross-sectional area of MN proximal to the entrapment site is shown (d) compared to normal MN (b).

Although the pathophysiology of CTS is still not completely understood, the increased pressure within carpal tunnel with sequel damage of the median nerve (MN) is generally accepted. The increased pressure can rise either from swollen flexor retinaculum (FR), flexor tenosynovium, and subsynovial connective tissue (SSCT) or interstitial fluid pressure within carpal tunnel. Change in interstitial fluid pressure was also associated with wrist position and both excessive extension and flexion of wrist could furtherly increase intra-carpal tunnel pressure [1]. Study also showed that MN can be dynamically squeezed between the flexor pollicis longus and flexor digitorum tendons when the wrist is flexed and the patient is holding a fist or flexing the fingers [5]. The damage of MN would trigger a series of degeneration and a segmental increase in the cross-sectional area (CSA) of the MN proximal to the entrapment site, as well as SSCT thickening and fibrosis was shown by ultrasonography (Figure 1) [6] These pathological changes furtherly result in adhesion and impaired longitudinal excursion [7] and transverse MN sliding [8]. Moreover, the fibrotic scar may encircle the MN accompanying with atrophy of nerve in chronicity of nerve entrapment [9].

The treatment of CTS can be divided into conservative and surgical intervention based on symptom severity. The conservative treatments are recommended initially for mild-to-moderate CTS. Although there are numerous conservative treatments, their effect is not significant and short-term. A recent review revealed 23% to 89% of patients treated by conservative treatments having poor outcome at 3 years' follow-up, and the surgical rate ranged from 57% to 66% in those patients [10]. Moreover, the newest systemic review only strongly supports therapeutic evidence in surgery, positioning, and corticosteroid injection. Bedsides, education, manual therapy, therapeutic exercises, or thermal treatments are conditionally supported only with low-to-moderate evidence [11]. Surgery is usually reserved for patients with moderate-to-severe CTS or patients having poor effect for conservative treatment. Although surgery is considered to have better mid- and long-term benefits than conservative managements, the surgical complications may occur, including surgical or pillar pain, inadequate release of the FR, adhesion, infection, nearby vessel injuries, or reduced grip strength [12].

## 2. Corticosteroid Injection for CTS

In the past few decades, corticosteroid injection under blind or ultrasound-guided was one of the mostly used conservative treatments for CTS with constructive effect. The underlying mechanism of corticosteroid for CTS is based on its anti-inflammatory and analgesic traits to reduce intracarpal pressure for symptom relief [13]. However, the systemic review only revealed its short-term effect, and the long-term effect of corticosteroid for CTS did not seem to be maintained [13, 14]. Bland [12] showed that 70% of patients treated with corticosteroid injection reach excellent improvement but 50% of patients relapse by one year. The study also revealed that 67.4% of patients ultimately undergo surgery after 12 months' postinjection with corticosteroid [15]. Moreover, recent trials show no dose-dependent effect of corticosteroid injection for CTS [16, 17], and 2 times of perineural injections of corticosteroid within 8 weeks did not provide further cumulative benefit compared with a single injection [18]. Furthermore, skin atrophy, depigmentation, localized erythema and warmth, subcutaneous atrophy, local infection, osteonecrosis, tendon rupture, and possible axonal and myelin degeneration are the potential harm related to corticosteroid injection [9, 19]. Although higher administered dosage of corticosteroid may have higher impact on the above adverse effects, there is no safe maximum amount of corticosteroid injections existing so far [19].

Moreover, corticosteroid injection is often mixed with local anesthetics to alleviate injection and postinjection pain, and lidocaine is the most commonly prescribed agent [16, 20, 21]. However, the local anesthetics can simultaneously cause blockade of sensory or motor conduction which may result in temporary motor weakness or potentially increase the risk of nerve injury by needle because patients might not aware of needle contact during injection. Likewise, as to neuronal toxicity, lidocaine has the highest clinically incidence of transient neurological symptoms related degenerative effect on myelin sheaths [22]. Systemic toxicity should also be concerned when injecting large volume (>20 mL) of lidocaine [9].

## 3. Novel Motor-Sparing Neural Injection for CTS

Recently, the novel motor-sparing neural injection in CTS with an inspirating effect and less adverse effects was published. These researches utilize an ultrasound-guided technique for the administration of normal saline (NS), 5% dextrose water (D5W), platelet-rich plasma (PRP), and hyaluronic acid (HA) without adding any analgesic agent. These novel injections potentially become alternative choice or the future mainstream method for CTS treatment with feasible for repetitive intervention.

### 3.1. Method of Ultrasound-Guided Neural Injection

**Figure 2:**
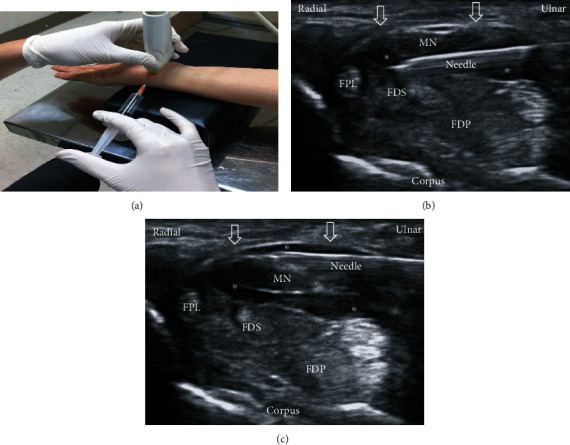
Ultrasound-guided short-axis injection image. (a) The position of in-plane short-axis injection at proximal inlet of carpal tunnel. (b) The short-axis view shows that the MN was separated from the subsynovial connective tissue and flexor tendon superficialis/profundus via hydrodissection (HD) (∗: injectate). (c) The short-axis view shows that the MN was separated from the flexor retinaculum (arrows) via HD (∗: injectate). MN: median nerve; FDS: flexor digitorum superficialis; FDP: flexor digitorum profundus; FPL: flexor pollicis longus.

**Figure 3:**
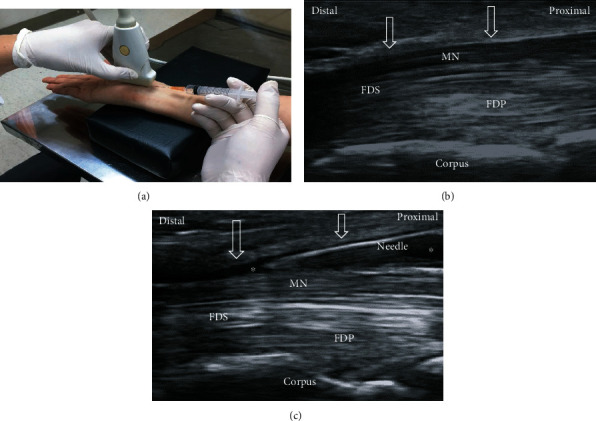
Ultrasound-guided long-axis injection image. (a) The position of in-plane long-axis injection advancing from the wrist crease to palm. (b) The long-axis view shows swollen nerve fascicles, flexor retinaculum (arrows), and inflamed tendons in same plane. (c) The long-axis view shows that the MN was separated from the flexor retinaculum (arrows) via HD. MN: median nerve; FDS: flexor digitorum superficialis; FDP: flexor digitorum profundus.

There are two most common methods of ultrasound-guided perineural injection for CTS, called the short- and long-axis approaches [23–27]. The short-axis technique employs an in-plane ulnar (Video 1) or radial approach at the proximal carpal tunnel inlet (Figure 2). The long-axis technique is employed using either the proximal-to-distal or distal-to-proximal (Video 2) direction of carpal tunnel (Figure 3). Short-axis injection was found to exhibit better hydrodissection effect (notable reductions in symptom and disability at 1-month postinjection) compared to the long-axis injection. This may be since the short-axis injection can simultaneously dissect FR and SSCT resulting in a greater hydrodissection effect between the SSCT and MN, because the adhesion and gliding resistance in these areas contribute to the prominent symptoms of CTS [28]. Additionally, operators using the long-axis scan face difficulties between swollen nerve fascicles, muscles, and inflamed tendons along the same long-axis plane; consequently, there is a concerning nerve trauma incidence although the long-axis approach has more contact area between FR and MN [29]. Further, the short-axis approach allows easier learning, improved injection accuracy, and lower nerve injury risk since the needle approach is parallel to the oval-shaped MN for nerve injury avoidance [27, 28, 30]. As both short- and long-axis HD were effective based on previous results, the intervention choice would depend on the operator's preference.

### 3.2. Types of Injectate for Neural Injection

#### 3.2.1. Normal Saline (NS)

Wu et al. [31] revealed the clinical efficacy of perineural injection with a single 5 mL NS for mild-to-moderate CTS compared with placebo group in a double-blind trial. In their study, the NS group showed significant symptom improvement at the 2^nd^ and 3^rd^ months' postinjection and significant decline in CSA of MN through the 1^st^ to 6^th^ months' postinjection. This study confirms the effect of nerve hydrodissection because hydrodissection can detach the entrapped MN from FR and SSCT to promote blood flow and decrease nerve compression injury for subsequent nerve regeneration [31–33]. However, the minimal required amount of injectate for hydrodissection effect is unknown yet and more clinical trial is needed in this filed.

#### 3.2.2. 5% Dextrose Water (D5W)

D5W was chosen for perineural injection, because its isoosmolarity would cause less irritation and is harmless for the nerve [34]. However, the exact therapeutic mechanism of D5W in treating CTS remains unclear. Inhibiting transient receptor potential vanilloid receptor-1 (TRPV-1) in blocking the release of substance P and calcitonin gene-related peptide (CGRP) was considered as the possible mechanism [33, 35]. Recently, perineural injection with D5W became a novel treatment for mild-to-moderate CTS according to series of high-quality clinical trials [36–39]. In 2017, Wu et al. [36] first proved six-month efficacy of the single ultrasound-guided perineural injection with 5 cc D5W for mild-to-moderate CTS without any adverse effect (control group: NS). In contrast to short-term effects of corticosteroid, that trend of outcomes reversed through the 3^rd^ to the 6^th^ month; the symptom and disability persistently improved with the longer follow-up in the D5W group and reached intergroup significance through the 4^th^ to 6^th^ month [37]. Lin et al. [39] showed that a larger volume of D5W provided better efficacy with respect to symptom and functional improvement for CTS with a 12-week postinjection follow-up. Likewise, the long-term effect of D5W injection has also be confirmed in Li's retrospective study in which 88.6% of 185 patients reported symptom relief more than 50% after a mean of 2.2 injections with a mean of 1–3 years' postinjection follow-up without any adverse effect [38]. Recently, Ho et al. [40] reported sensory nerve conduction velocity of the MN was found to be an independent prognostic factor (odds ratio: 1.201) of poor outcome postperineural injection with D5W.

#### 3.2.3. Platelet-Rich Plasma (PRP)

Platelet-rich plasma (PRP), also known as autologous concentrated platelets, contains numerous bioactive factors including transforming growth factor-*β*, platelet-derived growth factor, and vascular endothelial growth factor, which could promote tissue repair and regeneration. Previous animal studies approved the benefit of PRP for nerve regeneration [41]. Since 2015, some studies have investigated the efficacy of PRP injection for CTS with positive results [42–50]. Even though the definite mechanism of PRP for CTS remains ambiguous, the therapeutic effect is thought to be derived from mechanical hydrodissection, biological effects of PRP for MN's surrounding tissue, and neuroregenerative effect for MN [50]. Malahias et al. [42] first reported a pilot study and revealed positive 3-month outcomes after perineural injection of PRP for mild CTS in 2015. Until 2018, the first double-blind placebo-controlled trial revealed a positive effect of PRP with the 3-month follow-up for mild-to-moderate CTS compared with NS injection [46]. When comparing to traditional therapy, PRP was shown to have better efficacy for 3 to 6 months in patients with mild-to-moderate CTS, compared with splint [43] or corticosteroid injection [44, 48], respectively. However, another recent two nonblinded trials with very short-term follow-up (<10 weeks) reported inconsistent benefit of additional 1 mL PRP in patients with mild CTS treating wrist splint [45, 47]. Trull-Ahuir et al. [49] shared a randomized, triple-blind trial and shown significant improvement of hand grip strength at 6^th^ weeks in patient receiving surgical release of FR compared with platelet-poor plasma injection. The double-blind placebo-controlled study in 2021 had shown one-year efficacy of single-dose ultrasound-guided perineural injection with PRP in patients with moderate-to-severe CTS compared with NS injection [50]. Recently, Shen et al. [51] revealed less body weight, decreased distal motor latency and CSA of the MN predict better outcome after perineural injection of PRP for moderate CTS in 3 to 6 months follow-up.

#### 3.2.4. Hyaluronic Acid (HA)

HA, a glycosaminoglycan, has various molecular weights which influence its structure and biological properties [52, 53]. The viscoelastic and antiadhesion characteristic of HA made it clinically applicable for postsurgical adhesion [53, 54]. Su et al. [55] first demonstrated that a single HA injection, compared to NS, has significant improvements of function and symptoms at the 2^nd^ week postinjection for mild-to-moderate CTS, although no significant difference in the electrophysiologic study and CSA. Although the possible mechanisms of HA on CTS may be the antiadhesion, anti-inflammatory, and nerve regeneration effects, the therapeutic effects mainly depend on the antiadhesion effects, which HA can dissect the MN from the surrounding connective tissue and improve nerve mobility [55, 56]. However, the efficacy of single HA on CTS is only in the short-term; further studies with larger sample size, comparing different molecular weights and volumes of HA, are required to verify its therapeutic efficacy.

### 3.3. Comparison of Different Injectates for Severe CTS

#### 3.3.1. Objectives

Although individual studies have demonstrated the effectiveness of neural injection on CTS using these injectates, no published clinical study has compared the clinical efficacy of these injectates. Furthermore, current studies mainly enrolled mild-to-moderate CT. Only a few patients with severe grades were enrolled, and these patients are important in the search for novel treatments in the presurgical stage. Hence, this retrospective study aimed at comparing the efficacy of these four injectates for severe CTS.

#### 3.3.2. Materials and Methods

*(1) Study design*. A formal letter of exemption allowing retrospective chart review was obtained from the International Cellular Medicine Society Institutional Review Board (ICMS-IRB). We reviewed consecutive outpatient charts in a musculoskeletal clinic between Jan 2018 and Dec 2020 for patients diagnosed with severe CTS who underwent ultrasound-guided neural injection of the MN to manage neuropathic pain or numbness and associated functional impairments in daily activities. Charts were consecutively reviewed to identify participants who had severe CTS and received MN injection only once, exclusively with NS, D5W, PRP (XCELL PRP, APEX Biologix, USA), or HA (Monovisc (4 mL, 22 mg/mL sodium hyaluronate, 1000-2900 kDa, DePuy Synthes, USA), with the use of lidocaine only for the placement of skin blebs. The duration of the symptoms, gender, and body mass index (BMI) of the patients were recorded, and 61 patients were available for analysis. The pros and cons of each injectate were clearly explained according to the currently available evidence. Informed consent was obtained from all patients to use their clinical information in anonymity for clinical evaluation and publications. Patients consented to be treated once and then observed for at least 6 months before they search for other treatments; however, they are allowed to continue physiotherapy once weekly, night splints (if they are already using) and oral medication, including Panadol and/or Gabapentin and oral supplements of vitamin B complex and vitamin D.

*(2) Inclusion and Exclusion Criteria*. The inclusion criterion was severe CTS for more than 6 months based on CSA > 15 mm^2^ [38, 57]; conservative treatments, including physiotherapy, night splints, and oral medications, failed in all patients; hence, they were allowed to choose from NS, D5W, PRP, and HA. The exclusion criterion was opting for other interventional treatments before 6 months' postneural injection, including dry needling.

*(3) Outcome Measurements*. All patients were assessed using the Boston Carpal Tunnel Questionnaire (BCTQ), which contains the symptom severity scale (SSS, 11 items) and functional status scale (FSS, 5 items); higher scores indicate greater severity, and only patients with average symptom and functional scores > 3 were included [58, 59]. Ultrasound images of the CSA of MN inside the carpal tunnel were assessed and recorded as in previous studies [36, 37]. The BCSQ scores and CSA were measured before injection and at the 1^st^ and 6^th^ months' postinjection.

*(4) Ultrasound-Guided Neural Injection*. All patients were treated by the same musculoskeletal physician with more than 11 years of experience in administering ultrasound-guided neural injections (GE Logiq S7, Domestic, USA, L3-12 linear transducer). An in-plane short axis to the MN ulnar approach was used in all patients. Each patient was given a skin entry point local anesthetic by injecting a skin wheel of 1% lignocaine using a 30 G 1-inch hypodermic needle; after that, the MN was hydrodissected using a 25 G 2-inch needle used to inject 6 mL of the chosen solutions (NS, D5W, and PRP) or 4 mL HA. Half the dose for each injection below and above MN was administered. MN was observed to be more rounded after the procedure and surrounded by a hypoechoic halo of fluid (Figure 2).

*(5) Statistical Analysis*. All data were statistically analyzed using IBM SPSS statistics version 22, and statistical significance was set at *p* < 0.05. The chi-square test and one-way ANOVA were used to analyze categorical or continuous demographic data, respectively, between groups. Repeated-measure ANOVA followed by a post hoc test was performed for intragroup data recorded at multiple time points. The one-way ANOVA followed by the Bonferroni post hoc tests were used for between-group comparisons.

#### 3.3.3. Results

In total, 61 patients met the recruitment criteria. Among them, 15, 16, 15, and 15 selected NS, D5W, PRP, and HA, respectively. The BCSQ scores were similar among all the four groups of patients before the treatment, and the patients were of similar age groups and body build (Table 1).

All patients reported minimal to no pain during and immediately after the procedures, and no adverse event was reported in any patient. None of the recruited patients complained of motor deficit after hydrodissection of the MN. At the 1^st^ and 6^th^ months after injection, patients in all groups reported significant improvements compared to baseline (all *p* < 0.001).

**Figure 4:**
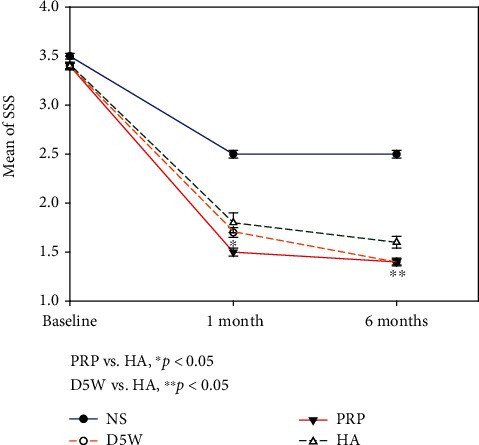
The symptom severity score (SSS) (mean ± standard error) between postinjection and baseline in the four groups at each time point. Patients in the 5% dextrose water (D5W), platelet-rich plasma (PRP), and hyaluronic acid (HA) groups have more significant improvement compared to those in the NS group at all measured time points. The progressive trend of improvement is PRP > D5W > HA, and intergroup differences reached significance at the 1^st^ month (PRP vs. HA) and the 6^th^ month (D5W vs. HA). SSS: symptom severity score; D5W: 5% dextrose water; PRP: platelet-rich plasma; HA: hyaluronic acid.

**Figure 5:**
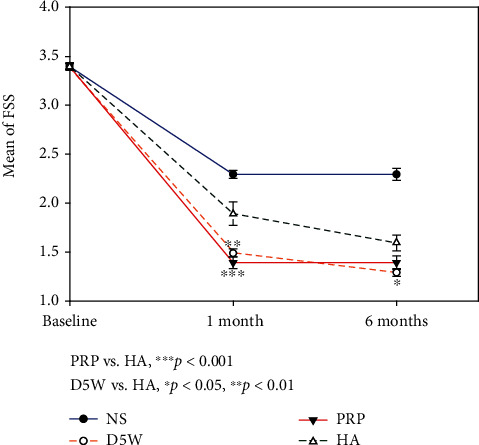
The functional status scale (FSS) (mean ± standard error) between postinjection and baseline in four groups at each time point. Patients in the 5% dextrose water (D5W), platelet-rich plasma (PRP), and hyaluronic acid (HA) groups have more significant improvement compared to those in NS groups at all measured time points. The progressive trend of improvement is PRP > D5W > HA except the 6^th^ month in which D5W was mildly better than the PRP group. The intergroup differences reach significance (D5W vs. HA on the 1^st^ and 6^th^ months; PRP vs. HA on the 1^st^ month). FSS: functional status scale; D5W: 5% dextrose water; PRP: platelet-rich plasma; HA: hyaluronic acid.

**Figure 6:**
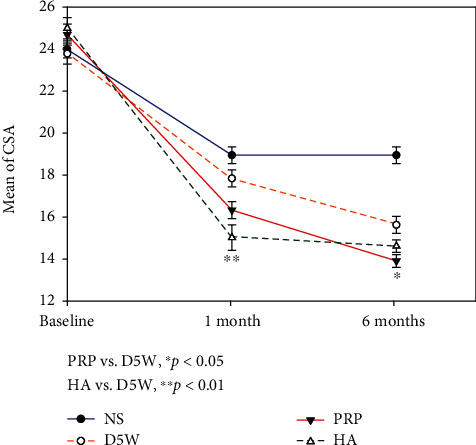
The cross-sectional area (CSA) (mean ± standard error) between postinjection and baseline in four groups at each time point. Patients in the 5% dextrose water (D5W), platelet-rich plasma (PRP), and hyaluronic acid (HA) groups have more significant improvement compared to those in NS groups through all measured time points except the 1^st^ month (D5W vs. NS). The progressive trend of improvement is HA > PRP > D5W on the 1^st^ month and reaches significance (HA vs. D5W). The improved trend is PRP > HA > D5W on the 6^th^ month, and statistical significance was observed (PRP vs. D5W). CSA: cross-sectional area; D5W: 5% dextrose water; PRP: platelet-rich plasma; HA: hyaluronic acid.

The NS group improved the least, with the D5W, PRP, and HA groups recording significant improvements compared to the NS group at all measured time points (all *p* < 0.05), except for CSA at the 1^st^ month between the NS and D5W groups (Figures 4, 5, and 6). For SSS, patients in the PRP group improved the most, followed by those in the D5W group, with those in the HA group recording the least improvement. The difference in SSS improvement reached significance on the 1^st^- (PRP > HA, *p* = 0.018) and 6^th^-month SSS (D5W > HA, *p* = 0.047) (Figure 4). Regarding FSS, the progressive trends between the three groups were similar to those of SSS, except for the 6^th^-month value between PRP and D5W, in which D5W was slightly better than PRP. Moreover, the intergroup difference in FFS improvement reached significance (D5W > HA on the 1^st^ and 6^th^ months, *p* = 0.002 and 0.016, respectively; PRP > HA on the 1^st^ month, *p* < 0.001) (Figure 5). The decrease in CSA difference was more pronounced in HA than in D5W and PRP (HA > PRP > D5W) at the 1^st^-month follow-up, reaching significance between the HA and D5W groups (*p* = 0.001). The difference in CSA improvement in the PRP group was more than that in the HA group (PRP > HA > DW) at 6^th^ months, and a statistical significance was observed in the PRP group compared to the D5W group (*p* = 0.012) (Figure 6).

#### 3.3.4. Conclusion

- Among the four neural injectates, single doses of PRP, D5W, and HA were more efficient than NS as regards symptomatic and functional improvement, as well as decreased CSA of MN, for severe CTS
- For symptom and functional improvement, single injections of PRP and D5W seemed more effective than that of HA within 6 months postinjection. Moreover, PRP and D5W had similar effects, although PRP was slightly better than D5W
- For reducing CSA, PRP and HA seemed more effective than D5W. HA was the most effective at the 1^st^-month postinjection (HA > PRP > D5W), and PRP was the most effective at the 6^th^-month (PRP > HA > D5W).

### 3.4. Recommendations for Neural Injectate Selection

The recommended selection of those neural injectate does not exist, and the decision seems based on the physician's choice. However, we advocate trying D5W firstly considering its cost effect based on a recent clinical trial, meta-analysis, and findings of above retrospective results [60, 61]. Although Shen et al. [60] reported that a single PRP injection was more effective in decreasing CSA of the MN at 3 and 6 months' postinjection compared to a single D5W injection, the differences of improvement in symptom and most electrophysiological studies did not reach statistical significance between groups. Moreover, recent network meta-analysis in 2020 shows that the D5W injection was likely to be the best treatment for symptom relief, followed by PRP and corticosteroid injection [61]. Our results also confirmed the findings mentioned above. Although PRP seemed slightly more effective than D5W and HA within 6 months, considering the cost-effectiveness, PRP seemed disproportionate to the progress of symptom relief for severe CTS. The recommended injection interval of D5W is 1 to 4 weeks according to prognosis and the average times of injection is around 3-5 times depending on outcome. If patient has poor or slow improvement after D5W injection, the PRP could been considered. If patients have persistent or recurrent symptom after surgery, the HA can been added based on possible obvious postsurgical adhesion of MN. Further other prospective researches with head to head or comparative study of these injectates in different grading CTS are required to establish the recommended protocol.

## 4. Future Recommended Study

Future studies should clarify several questions regarding these novel motor-sparing neural injections for CTS.

- Their definite pathophysiological and pharmacological effect on CTS
- Whether there is a cumulative effect of these injectates or not
- The optimal dosage and frequency of these injections
- The effect of these injections for subgroup patients who have higher risk for developing with CTS, e.g., uremia, diabetes mellitus, and rheumatoid arthritis
- How about the clinical effect of these injections in patients with surgery failure or postsurgery recurrence?

## 5. Conclusions

These motor-sparing neural injections for CTS can prevent motor weakness or potential neuronal toxicity of CTS patients. Moreover, some of these neural injections were proven to be more effective than traditional conservative managements. The D5W, PRP, and HA proved to be more effective than NS for severe CTS in our study, and their effects kept improving within 6 months after only a one-time injection. Moreover, PRP and D5W seemed more effective than HA in the improvement of symptoms and function. Bedsides, PRP and HA seemed more effective than D5W in reducing the CSA of MN. More large-scale prospective randomized control trials are needed to compare these solutions in different CTS grades. Finally, those motor-sparing neural injections are not intend to replace surgery. If patients have poor effect after those managements, the surgical indication should be recommended.

## Figures and Tables

**Table 1:** Baseline demographic and clinical characteristics of study patients.

	NS group (*n* = 15)	D5W group (*n* = 16)	PRP group (*n* = 15)	HA group (*n* = 15)	*p* value^a^
Age (year) (SE)	54.8 ± 1.2	54.9 ± 1.2	54.9 ± 1.2	54.1 ± 1.0	0.957
BMI (kg/m^2^) (SE)	27.7 ± 0.5	27.8 ± 0.4	27.7 ± 0.5	27.9 ± 0.4	0.996
Gender					0.998
Male (*n*) (%)	7 (46.7)	7 (43.8)	7 (46.7)	7 (46.7)	
Female (*n*) (%)	8 (53.3)	9 (56.3)	8 (53.3)	8 (53.3)	
Duration(months) (SE)	13.3 ± 0.6	13.3 ± 0.8	14.0 ± 0.7	13.8 ± 0.3	0.844
SSS (SE)	3.5 ± 0.03	3.4 ± 0.04	3.4 ± 0.03	3.4 ± 0.03	0.663
FSS (SE)	3.4 ± 0.03	3.4 ± 0.03	3.4 ± 0.04	3.4 ± 0.04	0.874
CSA (mm^2^) (SE)	23.9 ± 0.4	23.7 ± 0.5	24.6 ± 0.5	24.9 ± 0.5	0.287

NS: normal saline; D5W: 5% dextrose water; PRP: platelet-rich plasma; HA: hyaluronic acid; SE: standard error; BMI: body mass index; SSS: symptom severity scale; FSS: functional status scale; CSA: cross-sectional area. ^a^One-way ANOVA or chi-square test.

## Data Availability

The data used to support the findings of this study are included within the article.

## Supplementary Materials

Supplementary Materials Video 1: the video shows the short-axis, in-plane, ulnar, and radial approaches of ultrasound-guided hydrodissection of the median nerve. Video 2: the video shows the ultrasound-guided long-axis approach of hydrodissection of the median nerve.
