# Supplementary material for: Novel Motor-Sparing Ultrasound-Guided Neural Injection in Severe Carpal Tunnel Syndrome: A Comparison of Four Injectates
Source: Biomed Res Int. 2022 Feb 17;2022:9745322. doi: 10.1155/2022/9745322 (PMC8872692; doi:10.1155/2022/9745322)
Supplement: Supplementary Materials — Video 1: the video shows the short-axis, in-plane, ulnar, and radial approaches of ultrasound-guided hydrodissection of the median nerve. Video 2: the video shows the ultrasound-guided long-axis approach of hydrodissection of the median nerve. [file 9745322.f1.docx]

**Supplementary material:**

**Video1:** [**https://www.dropbox.com/s/7uyp8171dbf8zhk/MH%20HD%20both%20approaches.mp4?dl=0**](https://www.dropbox.com/s/7uyp8171dbf8zhk/MH%20HD%20both%20approaches.mp4?dl=0)

**Legend: Video shows the short-axis, in-plane, ulnar and radial approaches of ultrasound-guided hydrodissection of median nerve.**

**Video 2.**

[**https://www.dropbox.com/s/9uchgjfwfskgsrp/MN%20HD%20SAX%20to%20LAX%20with%20Name.mov?dl=0**](https://www.dropbox.com/s/9uchgjfwfskgsrp/MN%20HD%20SAX%20to%20LAX%20with%20Name.mov?dl=0)

**Video shows the ultrasound-guided long-axis approach of hydrodissection of median nerve.**
